# Integrating Art and Science in Undergraduate Education

**DOI:** 10.1371/journal.pbio.1001491

**Published:** 2013-02-26

**Authors:** Daniel Gurnon, Julian Voss-Andreae, Jacob Stanley

**Affiliations:** 1 Department of Chemistry and Biochemistry, DePauw University, Greencastle, Indiana, United States of America; 2 Professional artist, Portland, Oregon, United States of America; 3 Department of Art, Lindenwood University, St. Charles, Missouri, United States of America

## Abstract

Weaving the visual arts into a science curriculum can both help develop scientific imagination and engage non-scientists.

The prevailing vision for undergraduate science education includes increased collaboration among teachers of science, technology, engineering and math (STEM) and an overhaul of introductory courses [1]–[4]. But by staying within the borders of STEM, are we overlooking connections between the arts and innovative science? Likewise, are we missing an important opportunity to inspire and inform nonscientists? Here we explore how weaving the visual arts into a science curriculum can both help develop scientific imagination and engage nonscientists. As an example, we describe a recent collaboration between artists and scientists to create a series of science-inspired sculptures.

## Creativity and Intuition in the Arts and Sciences

Innovative science has long been linked with creative pursuits. In a more-than-century-old address entitled “The Power of Imagination in Science" [5], Jacobus Hernicus van't Hoff listed several highly successful scientists who were also poets, artists, or writers of fiction, including Galilei, Newton, and Faraday. Later awarded the first Nobel Prize in Chemistry, van't Hoff himself idolized Lord Byron and wrote poetry [6]. The list has grown considerably since van't Hoff's day, raising the question of whether exercising creativity through art *contributes* to the success of innovative scientists. Indeed, a recent study found Nobel laureates are more likely to pursue artistic endeavors than are members of the Royal Society and National Academy of Sciences, who are in turn more artistically engaged than the “average" scientist [7]. A separate review of the careers of a few dozen scientists found that while few practiced the visual arts, those who did tended to publish high-impact, highly cited research [8].

The fact is artists and scientists are more alike than typically portrayed. Both share an irresistible drive to describe and interpret our experiences, motivated frequently by, as van't Hoff put it, “the pursuit of an idea which exists only in the mind…and represents the result of imagination" [5]. The artist turns passionate explorations of the wonderful into works of art, and the scientist translates them into words and equations; but what drives innovation in science is inseparable from the elemental urge to express ourselves artistically. In Albert Einstein's words, “the greatest scientists are artists as well" [9]. Einstein believed his insight, like that of an artist, came more from intuition than from intellectual reasoning [10]. Other successful scientists count intuition, defined as “instinctive knowing without the use of rational processes," as an important component of scientific discovery [11]. Perhaps one of the reasons Nobel Prize–winning scientists are almost three times more likely to have an arts and crafts avocation [7] is because intuition is so central to the artistic process.

## Inspiring with Art and Science

Could art instruction help produce more innovative scientists? At present, support for the educational benefits of art-science partnerships is anecdotal. There have been surprisingly few attempts to test the widely held assumption that studying the arts makes one more creative in general [12]. And while the visual arts can develop students' creativity, objectivity, perseverance, spatial reasoning, and observational acuity—all key skills in science—it is not clear whether skills developed through artistic pursuits can transfer to other fields [12]–[14]. Nevertheless, there are compelling reports of collaborations at the K–12 and professional levels that have enriched not just audiences but also the scientists and artists at the center of the work [15],[16]. These projects suggest that combining art and science can have transformative effects. Indeed, the lingering question of knowledge transfer is all the more reason to develop projects where the boundaries between art and science are blurred. As with the teaching of literacy alongside scientific thinking [17], merging two traditionally separate subjects can yield unexpected rewards.

The benefits of art-science collaboration also come from the product itself, which holds potential to inspire nonscientists. Reports on science education emphasize the importance of providing a solid foundation in science for all students, but considering many college students never complete more than a single science course, providing a *lasting* foundation through traditional classroom instruction is an impossible task. For students who view science as just another checkbox on a long list of graduation requirements, we must find creative ways to cultivate a lasting sense of wonder and curiosity for scientific discovery. There are, after all, many online resources dedicated to providing world-class science education free of charge. By inspiring curiosity, a specialty of art-science collaborations, we provide motivation to learn. As science writer Philip Ball put it, “that's what good ‘sciart’ does: rather than seeking to educate, it presents some of the textures of science in a way that nudges the mind and enlivens the senses" [18].

## *Villin*: An Undergraduate Art-Science Collaboration

At DePauw University, the annual ArtsFest event presented an opportunity for collaboration between students and faculty from the chemistry and sculpture departments. The project was connected to a class called Introduction to Research, offered to first- and second-year students in DePauw's Science Research Fellows program. Although the design of the course varies by instructor, each iteration introduces aspects of scientific research that can be challenging to convey in a traditional classroom setting. In this particular iteration, our goal was to emphasize the importance of imagination and metaphor in understanding and communicating modern science [19]–[21]. At the beginning of the semester, the class of five students met with the biochemistry professor (DG) for a three-hour class each week, blending a crash course in protein structure with group discussions of primary literature. By coordinating visits to an introductory sculpture class (taught by JS) held in the same time bank, we were able to teach the students fundamental concepts of technique and design, and how to critique visual art. Later in the semester, the students and faculty teamed up with a professional artist (JV-A) to create a sculpture inspired by protein-folding research.

**Figure 1:**
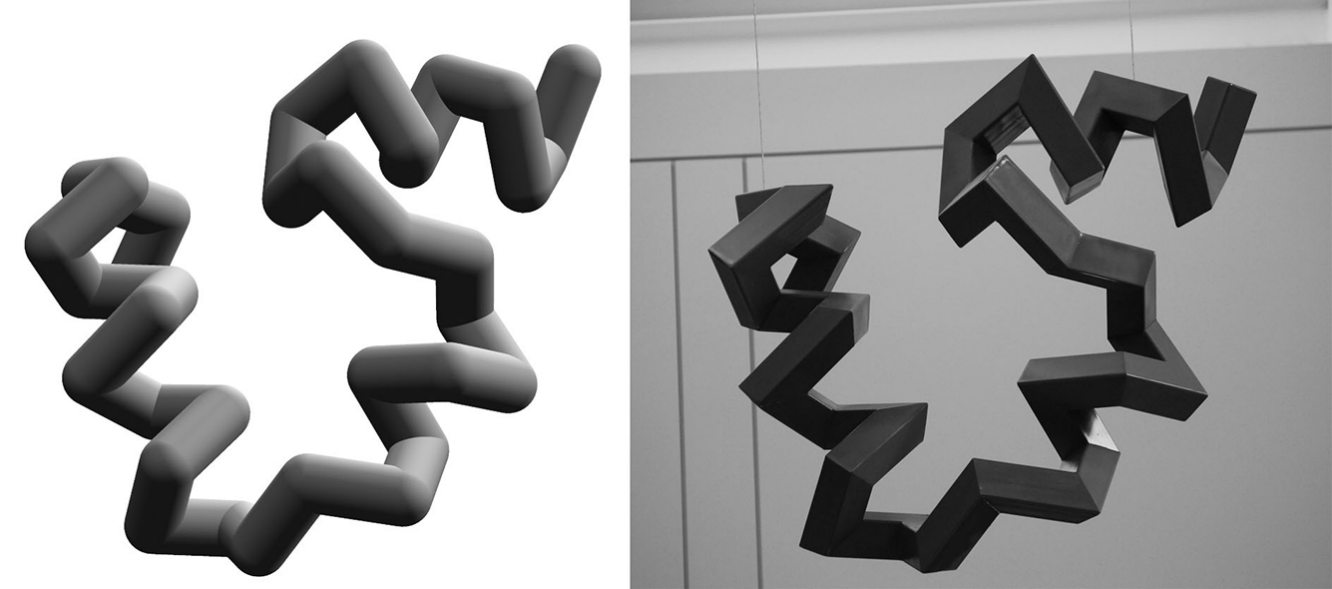
With the students' help, we transformed atomic coordinates (*left*, rendered with UCSF Chimera[28]) into remarkably accurate three-dimensional steel sculptures (*right*, approximately 1 m in height).

**Figure 2:**
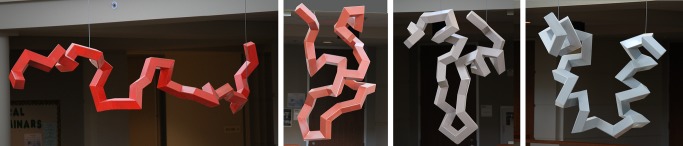
Each component of the sculpture represents a snapshot of a protein as it contorts from open chain (red) to native fold (gray).

**Figure 3:**
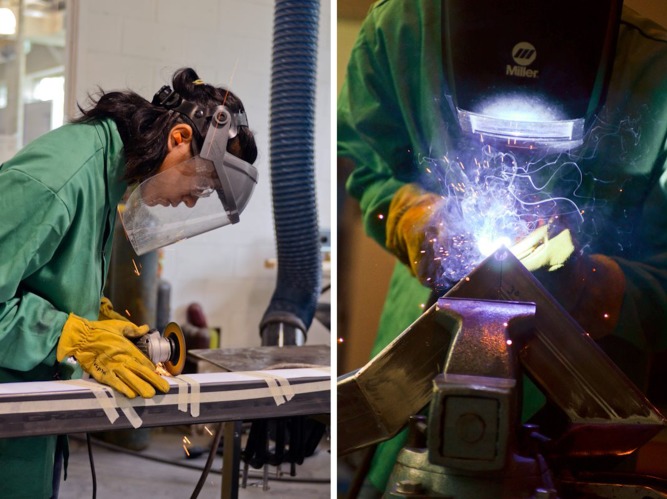
Undergraduates helped design and fabricate steel protein sculptures using published scientific data. (Photos by Larry Ligget.)

*Villin* is composed of four twisting steel structures. Each component of the sculpture represents a snapshot of a protein as it contorts from open chain to native fold. To create the sculpture, we transformed coordinates from a molecular dynamics trajectory of the villin headpiece domain [22] into precise miter-cut templates, then applied them to 3-inch (80 mm) square steel tubing. Individual “amino acid residues" were excised with angle grinders and welded into three-dimensional shapes through a process akin to assembling the angular corners of a picture frame (Figures 1–3). The design relies on a method developed by artist Julian Voss-Andreae to accurately portray protein backbones as miter-cut objects [23]. A former scientist himself, Voss-Andreae has used this technique as a foundation for several of his sculptures [24]–[26], among them *Angel of the West*, a 12-foot-tall human antibody commissioned for the Scripps Research Institute [27].

Although plans to use Voss-Andreae's construction techniques were set before the semester began, several design aspects were determined with student input. As students learned about protein structure and protein folding, we discussed whether any important ideas—such as the concept of an energy landscape—could inform the sculpture design. Students built wooden maquettes and used the software package UCSF Chimera [28] to aid in visualizing their ideas. Of all of our discussions, which included installation, lighting, and even slicing symbols into the steel beams with a plasma cutter, the use of color generated the most debate. Some students wanted a jumble of colors representing as many features of the protein as possible, while others wanted a more simplified design. Eventually the group settled on a monochromatic palette, with energy represented by color saturation: the unfolded chain in red, the folded protein in gray. The decision came partly from consideration of the artist's prior work, and also from primary literature on color perception that the students themselves sought out (for example, [29]). At the semester's end, the students presented the work to their peers using metaphors they developed to convey the enormous differences in physical and temporal scale—helping to make the art, and the science, more meaningful.

## Art-Science Sculpture as a Versatile Teaching Tool

*Villin* provides an example of the educational and communicative power of combining aesthetic design with scientific knowledge. For one thing, the installation attracts a diverse audience—but more importantly, it has a way of encouraging people to think differently. Art classes visit the structures to discuss public art and three-dimensional design. Students puzzle over the underlying system for the complex joints; to these art students, the project is a contemporary sculpture, drawing on the history of minimalism and on abstract works like Constantine Brancusi's *The Endless Column*. Yet the sculptures also spark interest in the underlying science; in one instance a student asked, “How do scientists *know* the angles in a protein?"

In contrast, biochemists see *Villin* as a representation of a protein backbone (the α-helices are hard to miss), and in a biochemistry course the piece encourages discussions of protein structure and dynamics. Yet *Villin* is unmistakably a work of art, and as such it resists the kind of straightforward analysis afforded to the physical models found in many classrooms. Discussions expand to include the strangeness of the molecular world compared to our own direct experience. We discuss visual strategies of conveying information and confront the multiple ways scientists have invented to represent molecular structure. Indeed, students are often surprised to learn that the textbook ribbon models of protein backbones are as much an abstraction of the molecular world as 400 lb. of welded steel, and to learn that color, as we experience it, does not exist in the molecular world.

For the science students who built the piece, the experience of fabricating the sculpture with their own hands provided a tactile insight into structures they were only accustomed to studying intellectually. Perhaps as a result, students developed an intuition for complex concepts of protein structure and folding [30]. For example, while constructing a wooden maquette of the most elongated backbone, students wondered whether a protein would begin folding as it emerges from the ribosome, and thus never truly resemble the completely unfolded structure they were building; in truth, the molecular dynamics simulation we employed begins with an artificially elongated molecule. On another occasion, walking alongside the row of completed structures, a first-year student asked if proteins fold by first crumpling inward and later adopting the recognizable patterns of α-helices and β-sheets—a question that is, in fact, still a matter of debate in the field [31].

We can offer some insights for those interested in designing a similar project: (1) Connecting a project to a campus-wide event provided excellent motivation, not only because it established a fixed deadline, but because students knew their work would be viewed by the community. (2) Meeting times for lab and studio art courses often conflict —this is partly why few science students take art courses, and vice versa—but the overlap provides an opportunity. In our case, schedule overlap between the science and sculpture classes allowed for efficient use of faculty time. (3) In addition to faculty and students' willingness to work long hours, student volunteers recruited from other art and science classes were vital to meeting our deadline. (4) Productive and lively discussions were facilitated by faculty and students working together in all aspects of the process, including construction. (5) Projects like *Villin* can be challenging to fund, but they can also be a good fit for more than one funding source. We were able to make ours work by combining resources from the science class budget and from money allocated for special events.
